# Design and Validation of a Multi-Modal Bioreactor System: Assessing the Effects of Perfusion and Cyclic Tensile Stimulation on Mechanical and Biological Properties of 3D-Printed Missing-Rib Auxetic Scaffolds

**DOI:** 10.3390/bioengineering13020140

**Published:** 2026-01-26

**Authors:** Tavila Sharmin, Sakhawat Hossan, Rohan A. Shirwaiker

**Affiliations:** 1 Edward P. Fitts Department of Industrial & Systems Engineering, North Carolina State University, Raleigh, NC 27695, USA; 2 Comparative Medicine Institute, North Carolina State University, Raleigh, NC 27695, USA; 3 Department of Computer Science, University of North Carolina at Greensboro, Greensboro, NC 27403, USA; 4 Bezos Center for Sustainable Protein, North Carolina State University, Raleigh, NC 27695, USA; 5 Lampe Joint Department of Biomedical Engineering, University of North Carolina at Chapel Hill and North Carolina State University, Raleigh, NC 27695, USA; 6 Department of Mechanical & Aerospace Engineering, North Carolina State University, Raleigh, NC 27695, USA

**Keywords:** bioreactor, perfusion, mechanical stimulation, auxetic scaffold, biofabrication

## Abstract

Bioreactors used for the maturation of cell-seeded tissue-engineered scaffolds should essentially mimic the dynamic in vivo environments experienced by the native tissues they intend to substitute. In addition to perfusion of growth medium to facilitate continuous mass transfer, application of appropriate mechanical stimulation is important to enhance cellular responses in scaffolds for tissues such as tendons, skin, and cardiac muscle that experience dynamic loading. This study focuses on the development of a multi-modal custom bioreactor capable of applying cyclic tensile stimulation and perfusion within physiologically relevant ranges while minimizing shear stress detrimental to cells seeded on scaffolds. To validate the bioreactor design and operation, we assessed the effects of tensile stimulation (0.1 Hz, 2000 cycles/day) and perfusion (media flow rate = 0.15 mL/min) over 21 days on the biofunctional performance of 3D-bioplotted polycaprolactone (PCL) auxetic scaffolds with a representative design (missing-rib pattern) characterized by negative Poisson’s ratio similar to the aforementioned soft tissues. The scaffold had a tensile yield strain of 9.14%, yield strength of 0.25 MPa, elastic modulus of 2.85 MPa, and ultimate tensile strength (UTS) of 1.32 MPa. The application of perfusion and tensile stimulation (0–5% cyclic strain) for 21 days did not adversely affect the yield strength and elastic modulus of the scaffold but affected its UTS (22.5% decrease) compared to the control cultured without perfusion or stimulation. Notably, it resulted in significantly improved fibroblast cellular responses (DNA = 29 µg/g sample and collagen = 371.78 µg/g sample) compared to the control (7.52 µg/g sample and 163.51 µg/g sample, respectively). These results validate the bioreactor system operation and the ability of multi-modal stimulation to control biofunctional responses of auxetic scaffolds, which will serve as the basis for future studies that will optimize auxetic scaffold design and dynamic culture parameters for NPR tissue-specific applications.

## 1. Introduction

Static in vitro cultures in tissue engineering exhibit significant limitations, primarily due to inadequate mass transfer, which results in non-uniform nutrient delivery and waste product accumulation [1,2]. This leads to constrained oxygen diffusion, causing hypoxic conditions within the interior regions of the construct [3]. Additionally, static cultures fail to provide the necessary mechanical stimuli for appropriate cellular function and differentiation, as they do not replicate in vivo mechanical cues relevant to various tissues [4,5]. These limitations can be mitigated by designing bioreactors that incorporate advanced functionalities including perfusion systems and mechanical stimulation mechanisms [5].

Perfusion bioreactors can closely simulate the dynamic in vivo environment by continuous mass transfer critical for the delivery of nutrients and oxygen and the removal of wastes, thereby improving cell viability and formation of more physiologically relevant tissue constructs [1,2,5,6,7,8]. Some degree of fluid shear stress induced by perfusion can act as a stimulus on tissue constructs, enhancing tissue-specific cellular responses [5], but optimizing the flow rates is essential to avoid detrimental impacts such as the removal of cells from the scaffold surface and essential paracrine factors [8].

In addition to perfusion, mechanical stimulation during in vitro culture can promote cell proliferation, differentiation, alignment, and functional maturation when engineering tissues that experience mechanical loading in vivo [4,5]. For example, tensile stimulation in a physiologically relevant range is beneficial for engineering tissues such as tendons, ligaments, muscles, and skin [9,10,11]; it can promote cell proliferation, extracellular matrix (ECM) formation, and ECM fiber alignment, thereby improving the biomechanical properties of the engineered tissue. Tissue engineering scaffolds to be subjected to tensile stimulation must possess appropriate mechanical properties, including elasticity, resilience, and toughness, to be able to sustain the applied cyclic load [12]. Electrospun scaffolds have generally offered these properties suitable for tensile stimulation [13,14] more favorably than 3D-printed scaffolds with typical designs (e.g., 0/0° or 0/90° strand laydown patterns) [15,16,17,18]. Three-dimensionally printed scaffolds with such conventional patterns are typically rigid and lack the geometry-driven structural elasticity to accommodate cyclic tensile deformation, often resulting in premature failure due to high stress concentrations at the filament junctions [19,20].

To address this, recent studies have considered design-induced elasticity in 3D-printed scaffolds by incorporating auxetic designs [20,21,22,23,24,25,26,27]. In general, auxetic designs can induce various metamaterial properties including negative Poisson’s ratio (NPR), enhanced elasticity, high strength-to-weight ratio, impact resistance, and fracture toughness [20,21,22,23,24,25,26,27,28,29,30,31]. These properties arise from the unique unit cell design of the auxetic structure rather than the scaffold material itself. As such, NPR properties have been documented for various native tissues, including tendons and skin [32,33]. Scaffolds with NPR also hold potential as engineered tissue patches for organs that naturally undergo expansion and contraction, such as heart, liver, and stomach [24,25]. Under tensile stimulation, auxetic scaffolds can expand both in longitudinal and lateral directions in response to the applied force due to their NPR, enabling more uniform stress distribution with reduced localized stress concentration across the scaffold [21,24,26,28,31]. In contrast, conventional 3D-printed scaffolds with positive Poisson’s ratio undergo lateral contraction, resulting in less uniform stress distribution [21,30,31]. Such deformation behavior enables auxetic scaffolds to effectively maintain porous architecture and mechanical integrity under tensile loading [20,22,24,25,26,27], making them well-suited for tensile stimulation applications mimicking the dynamic biomechanical environment of load-bearing soft tissues.

Auxetic scaffolds have been studied for engineered skeletal muscle [23] and cardiac muscle [25] but under static culture conditions. Chen et al. (2020) [26] and Lee et al. (2022) [27] were the first to examine auxetic scaffolds under tensile stimulation for nerve and ligament tissue engineering, but their bioreactor did not employ media perfusion, which is important for facilitating nutrient diffusion and waste removal.

To address the aforementioned gaps in tissue engineering science and technology, this study focused on the design and validation of a custom perfusion bioreactor system capable of applying tensile stimulation. To validate the bioreactor operation, auxetic scaffolds with a missing-rib pattern were employed as a model to investigate the effects of the resulting in vitro dynamic conditions on their biofunctional properties. While multi-modal commercial bioreactors are available, they are often expensive and exhibit limitations, including inadequate adaptability for 3D scaffolds, limited ability to accommodate scaffolds of varying sizes and quantities, and limited control over applied mechanical strain [14,34,35,36]. Custom bioreactors such as the open-access design presented herein can overcome these challenges and help the broader research community advance the development of biomimetic engineered tissue technologies for faster and more effective clinical impact.

## 2. Materials and Methods

**Figure 1:**
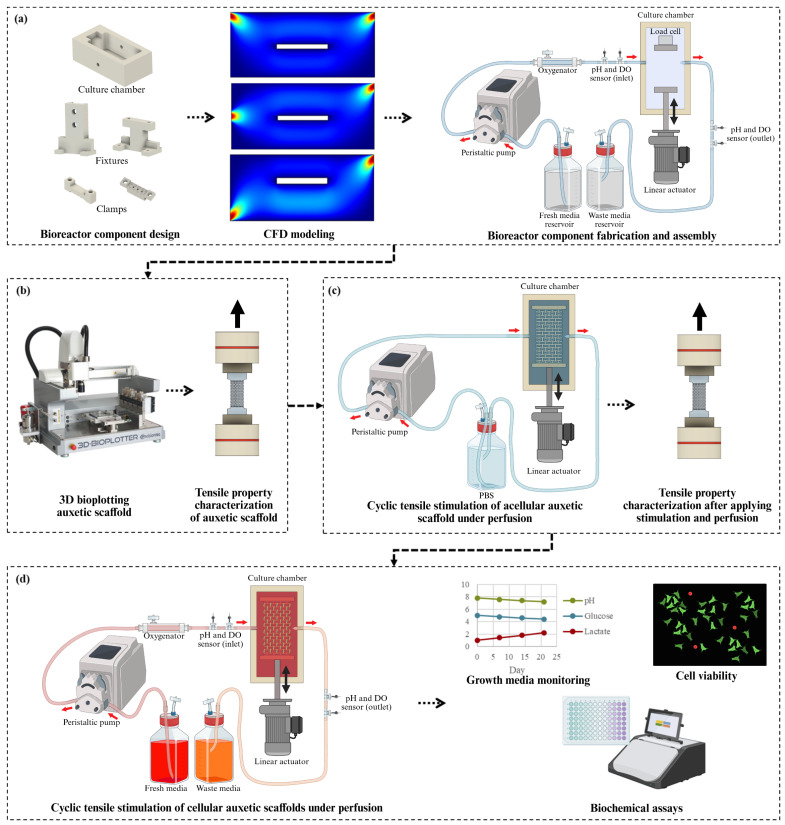
Study design for developing a custom perfusion bioreactor with cyclic tensile stimulation mecha-nism: (**a**) bioreactor component design, CFD modeling to identify optimal inlet/outlet configuration, component fabrication, and assembly, (**b**) 3D bioplotting auxetic scaffold (image courtesy of Envi-sionTEC, Gladbeck, Germany) and monotonic tensile property characterization, (**c**) characterizing effects of perfusion and cyclic tensile stimulation on tensile fatigue properties of acellular auxetic scaffold using the bioreactor, and (**d**) validation of the effectiveness of the simultaneous application of perfusion and tensile stimulation on cellular responses. Created in BioRender. Sharmin, T. (2026) https://BioRender.com/wvpeuh6 (accessed on 24 January 2026).

The overall study design is illustrated in Figure 1. First, we developed a custom perfusion bioreactor capable of applying simultaneous tensile stimulation within the physiological range to scaffolds, ensuring minimal shear-induced damage to the cell-seeded constructs using computational fluid dynamics (CFD) modeling. Next, we determined the baseline monotonic tensile properties of 3D-bioplotted polycaprolactone (PCL) scaffolds with the missing-rib pattern as a representative model. The missing-rib pattern is one of the most versatile auxetic patterns discussed in the literature, with its NPR and mechanical properties ranging within those relevant for various soft tissues [20,24,25]. Finally, we studied the effects of tensile stimulation (within the elastic limit determined from the monotonic tensile testing) and perfusion on fatigue properties of the 3D-bioplotted auxetic scaffolds and cellular responses in culture.

### 2.1. Design and Fabrication of Multi-Modal Perfusion–Tensile Stimulation Bioreactor System

#### 2.1.1. Key Bioreactor Components

**Figure 2:**
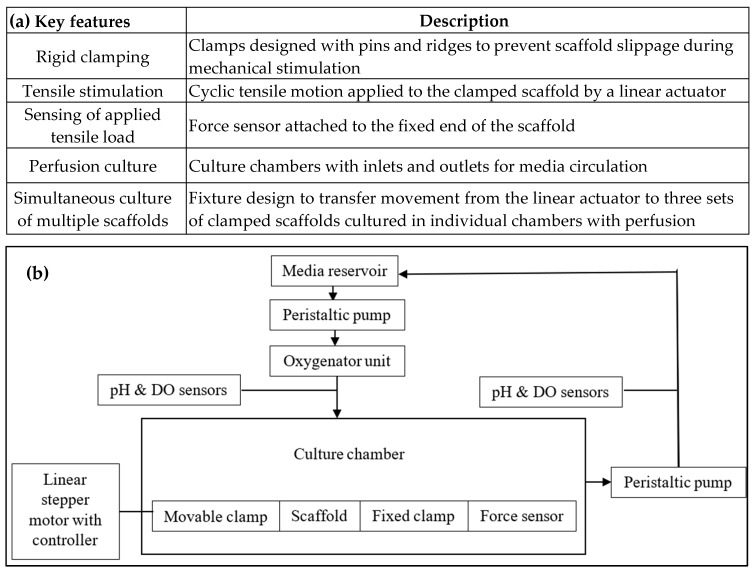
(**a**) Key features and (**b**) the schematic of the perfusion bioreactor system with a cyclic tensile stimulation mechanism.

The key features and schematic of the assembled bioreactor system enabled with perfusion and cyclic tensile stimulation are summarized in Figure 2. The required components (i.e., clamps, fixtures, and culture chamber) were designed in Autodesk Fusion 360 (education version, Autodesk Inc., San Francisco, CA, USA). The clamps and fixtures were fabricated using acrylonitrile butadiene styrene (ABS) via extrusion 3D printing (Stratasys F120, F370, Stratasys Ltd., Minneapolis, MN, USA). The culture chambers were CNC machined (HAAS VF2, Oxnard, CA, USA) using FDA-grade polycarbonate (Plasti-Block^®^, Omachron Plastics Inc., Ontario, Canada) based on the design informed by the CFD analysis presented in Section 2.1.2. The bill of materials with specifications of the other commercially procured components is provided in Table A1.

#### 2.1.2. CFD Modeling to Inform Optimal Chamber Design

**Figure 3:**
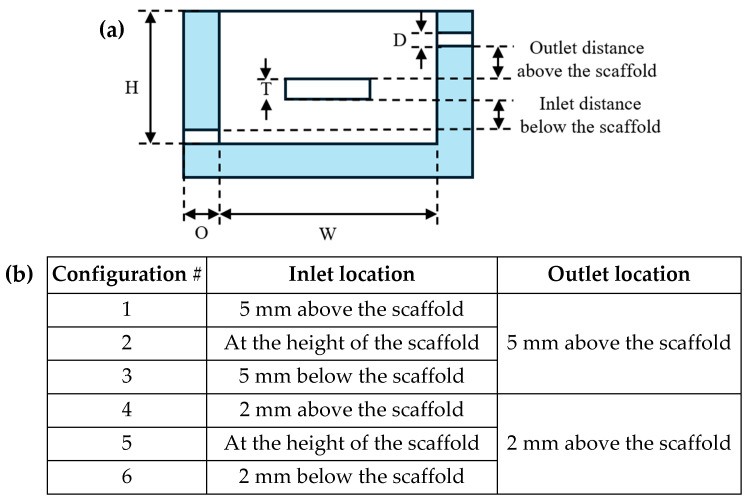
(**a**) Sectional view of the culture chamber illustrating the inlet/outlet locations from the scaffold (scaffold thickness, T = 2 mm; inlet/outlet diameter, D = 1.75 mm; culture chamber inner height, H = 25 mm, inner width, W = 35 mm, wall thickness, O = 8 mm) and (**b**) list of inlet/outlet configurations investigated using CFD models.

The overall culture chamber inner dimensions were set to L × W × H = 70 × 35 × 25 mm^3^ to enable sufficient room for installation of the other culture chamber components and ingress and egress of scaffolds (maximum size = L × W × H). CFD analysis was performed to determine the optimal configuration of the culture media inlet and outlet in the chamber to maximize the media circulation for an adequate supply of glucose and oxygen to the cell-seeded scaffolds while minimizing the flow-induced wall shear stress (WSS) on the scaffold surface, which can be detrimental to cells. CFD models were analyzed for six different configurations of the media inlet and outlet (Figure 3) using water as the model fluid (density = 1000 kg/m^3^ and viscosity = 0.001 kg/ms) in SOLIDWORKS (2023, SOLIDWORKS corp., Waltham, MA, USA). A laminar flow rate of 0.15 mL/min (lower bound of the peristaltic pump) was applied to the inlet of the culture chamber, while a zero-gage pressure was applied to the outlet. A no-slip condition was imposed on the bioreactor chamber, and the scaffold surface was considered hydrophilic. The inlet/outlet configuration that resulted in the lowest average shear stress was selected for fabrication.

### 2.2. PCL Auxetic Scaffold Design and 3D Bioplotting

**Figure 4:**
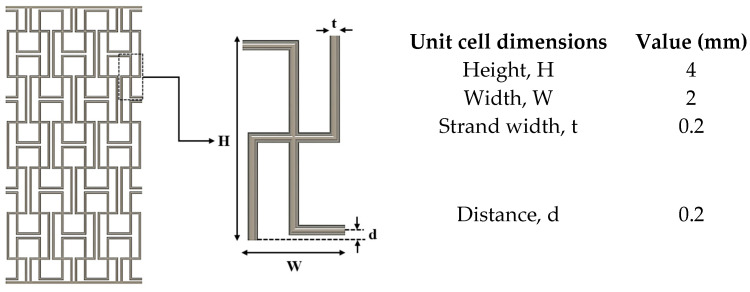
CAD model of the PCL auxetic scaffold (gage: 24 × 12 × 2 mm^3^).

The auxetic scaffold (gage: 24 × 12 × 2 mm^3^) with a missing-rib pattern was designed in Autodesk Fusion 360 as per the unit cell dimensions in Figure 4 and printed on a 3D Bioplotter (EnvisionTEC, Gladbeck, Germany) using a high-temperature printhead with nozzle Ø = 0.2 mm. The STL file was sliced into 0.16 mm layers (layer height = 80% of nozzle Ø as per the manufacturer’s recommendations and the literature [37,38]) and positioned on the bioplotter stage in the PerfactoryRP software (EnvisionTEC). The file was then preprocessed in VisualMachines (EnvisionTEC), and appropriate PCL bioplotting parameters informed by the literature [37,38,39] were assigned (temperature = 120 °C, extrusion pressure = 0.6 MPa, and speed = 1.2 mm/s). PCL pellets (M_w_ = 37 kDa, Polysciences Inc., Warrington, PA, USA) were preheated at 120 °C for 15 min before printing. To improve hydrophilicity, the bioplotted scaffolds were submerged in 50 mL of 2.5 M NaOH at 50 °C for 1 h and washed three times in deionized (DI) water. Prior to the mechanical and biological characterization, all scaffolds were sterilized by submerging in 70% EtOH for 2 h, then washing thrice in sterile phosphate-buffered saline (PBS) (Sigma-Aldrich, St. Louis, MO, USA) and air-drying under sterile conditions.

### 2.3. Baseline Tensile Testing of Scaffolds

**Figure A1:**
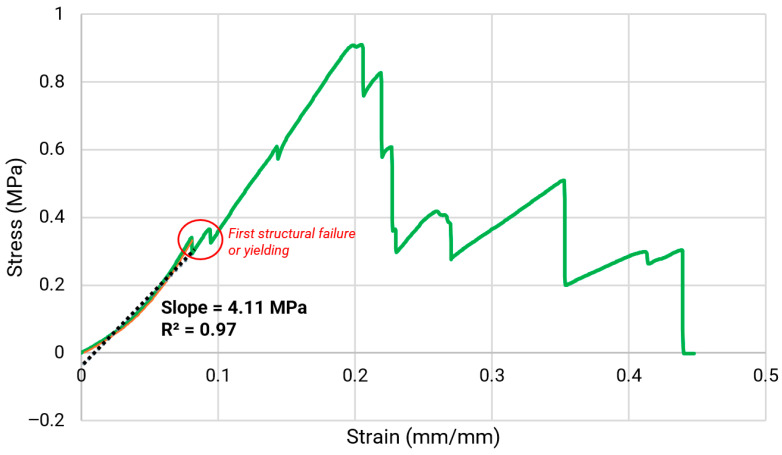
Representative engineering stress–strain curve (green) obtained from uniaxial tensile testing of one of the experimental samples. Mechanical properties were determined from the initial elastic region, defined as the portion of the curve prior to the first structural instability and yielding. The elastic modulus of the scaffold was calculated by performing linear regression on the stress–strain data within this region using Microsoft Excel (black dotted line), and a high coefficient of determination (R^2^) was required to confirm linearity and the validity of the modulus calculation. Yield strength was determined using a 0.2% strain offset method (red line), as indicated by the orange line. The maximum stress recorded during tensile loading was noted as the ultimate tensile strength (UTS).

**Figure A2:**
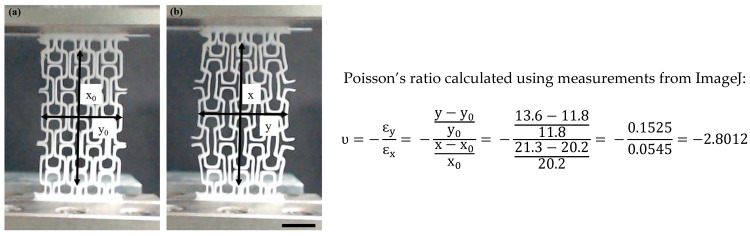
Poisson’s ratio calculation for a representative tensile tested experimental sample: (**a**) scaffold before tensile testing and (**b**) scaffold during linear-elastic deformation. Measurements were obtained via ImageJ analysis of images captured by a camera mounted on the UTM (scale bar = 5 mm) and used to calculate Poisson’s ratio using the standard formula.

The scaffolds (n = 3, a sample size consistent with other scaffold tissue engineering studies in the literature [24,31,40,41,42,43,44,45,46,47]) were subjected to monotonic tensile load in a Universal Testing Machine (UTM) (Instron, Norwood, MA, USA) using a 500 N load cell, 0.01 N preload, and 0.1 mm/s displacement rate until failure. From the force–displacement data, engineering stress–strain curves were generated to characterize the mechanical properties of the missing-rib scaffolds. The tensile elastic modulus of the scaffold was determined from the slope of the initial linear–elastic region prior to the first structural instability or yielding (see Figure A1) [48,49]. Yield strength was calculated using the 0.2% strain offset method. The ultimate tensile strength (UTS) was defined as the maximum stress recorded during tensile loading prior to the onset of progressive structural failures. Poisson’s ratio (PR) was calculated as the negative ratio of transverse strain to longitudinal strain within the same initial linear–elastic region used for modulus determination [22,24,50]. Transverse and longitudinal strains were calculated from images captured by a camera mounted on the UTM and analyzed using ImageJ (version 2.14.0/1.54f, National Institutes of Health, Bethesda, MD, USA) [51] (see Figure A2).

### 2.4. Assessing the Effects of Perfusion and Cyclic Tensile Stimulation on Scaffold Fatigue Properties

**Figure A3:**
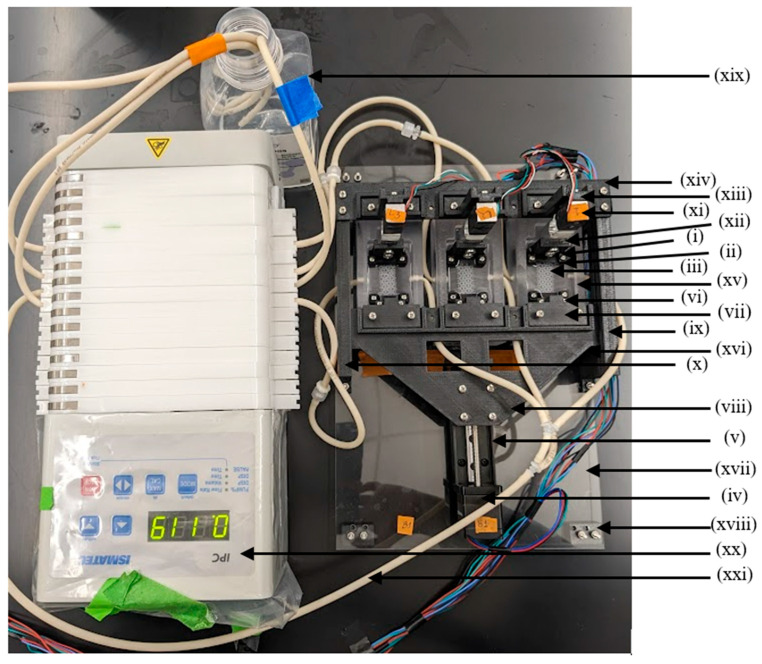
Representation of bioreactor setup for assessing the effects of perfusion and tensile stimulation on tensile fatigue properties of acellular missing-rib auxetic scaffold: (i) top clamp, (ii) bottom clamp, (iii) scaffold, (iv) linear actuator, (v) actuator support base (vi) fixture-1 actuator side, (vii) fixture-2 actuator side, (viii) movable plate, (ix) right-side guideway, (x) left-side guideway, (xi) force sensor, (xii) fixture-1 force sensor side, (xiii) fixture-2 force sensor side, (xiv) stationary plate, (xv) culture chamber, (xvi) base plate for culture chambers, (xvii) bioreactor mounting plate, (xviii) pedestal, (xix) PBS reservoir, (xx) peristaltic pump, and (xxi) tube.

The scaffolds (n = 3) were independently cultured (37 °C, 5% CO_2_) in PBS in the bioreactor under perfusion and cyclic tensile stimulation (Table 1) for 21 days (see Figure A3 for experimental setup). The maximum level of applied strain (5%) was set below the yield strain (9.14%, as determined via Section 2.3) to prevent permanent deformation during cyclic stimulation. Furthermore, 5% strain is within the levels at which NIH 3T3 cells (used in Section 2.5) have been reported to exhibit enhanced proliferation and ECM formation in non-auxetic scaffolds [52]. For the testing, one end of each scaffold was connected to the linear actuator, while the other end was attached to a force sensor. The tensile stress experienced by each scaffold sample at 5% strain over 2000 cycles/day was determined in situ using data from the load cells. Scaffolds cultured without perfusion and stimulation served as the static control group (n = 3). After 21 days, all scaffolds were tested in the UTM under monotonic tensile loading until failure, as per Section 2.3.

### 2.5. Assessing the Effects of Perfusion and Cyclic Tensile Stimulation on Cellular Responses

Cell-seeded scaffolds were assessed over 21 days of culture under four distinct culture conditions in the bioreactor system: perfusion + stimulation, stimulation only (no perfusion), perfusion only (no stimulation), and static control (no perfusion or stimulation). Each scaffold (n = 3/group) was seeded with 1 × 10^6^ NIH 3T3 cells (CRL-1658, ATCC, Manassas, VA, USA) in 300 µL of growth media (89% Dulbecco’s Modified Eagle Medium, 10% fetal bovine serum, 1% antibiotic–antimycotic, Thermo Fisher Scientific Inc., Waltham, MA, USA) and cultured (37 °C, 5% CO_2_) for 4 h in 60 mm Petri dishes. Then, the scaffolds were transferred to new Petri dishes and cultured in 8 mL of growth media for 3 days. The cell seeding efficiencies after 4 h were determined by counting the number of remaining cells in the Petri dishes used for cell seeding. After 3 days of static culture, scaffolds (all four groups) were transferred to the bioreactor and cultured over 21 days under their assigned condition—with or without cyclic tensile stimulation (0–5% strain, 2000 cycles/day, 0.1 Hz,) and/or perfusion flow of growth medium (flow rate = 0.15 mL/min, media volume in chamber = 33 mL) with continuous oxygenation using a hollow fiber silicone membrane gas exchanger. Growth media was refreshed every 7 days for all four groups, and cell metabolic activity was monitored by assessing the glucose and lactate concentration in the media once every 3 days using Cedex Bio HT Analyzer (Roche CustomBiotech, Penzberg, Germany). Cell viability was assessed via a Live/Dead assay (Thermo Fisher Scientific Inc.) on days 1 and 21 of bioreactor culture. The scaffolds were imaged (DMi8, Leica Microsystems, Wetzlar, Germany) and % viability was quantified (n = 5 images/scaffold) using ImageJ. After day 21, the scaffolds were prepared for biochemical assays to assess DNA, collagen, and sulfated glycosaminoglycan (sGAG) content. This 21-day terminal endpoint was selected to evaluate the capacity of the bioreactor system to support sustained cell viability and matrix deposition under continuous multi-modal stimulation, a timeframe consistent with foundational bioreactor validation and scaffold characterization studies [53,54]. In brief, scaffolds were sectioned, lyophilized for 48 h, and digested overnight in papain from papaya latex (Sigma-Aldrich) at 60 °C. A PicoGreen assay (Thermo Fisher Scientific) was performed for DNA quantification. A hydroxyproline (OHP) assay (Sigma-Aldrich) and a 1,9-dimethylmethylene blue (DMMB) assay (Sigma-Aldrich) were performed to assess collagen and sGAG content in the samples. The details of these assays are provided in Table A2. The total DNA, collagen, and sGAG content were normalized to the mass of the scaffolds.

### 2.6. Statistical Analysis

The effects of perfusion and/or cyclic stimulation on scaffold tensile properties and cellular responses were analyzed via one-way ANOVA and Tukey’s HSD post hoc tests using RStudio (version 4.2.1, RStudio Inc., Boston, MA, USA) to determine significant differences between groups (α = 0.05).

## 3. Results and Discussion

### 3.1. Multi-Modal Perfusion–Tensile Stimulation Bioreactor System Design

**Figure 5:**
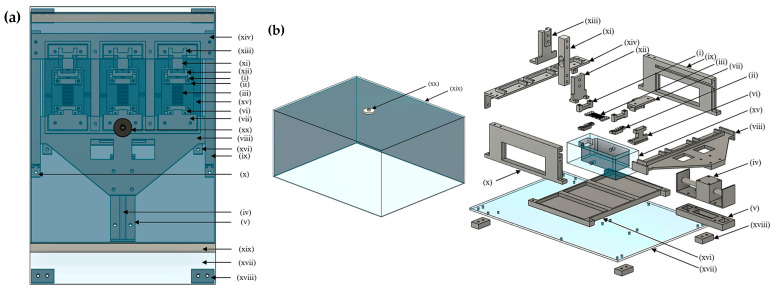
CAD model of the bioreactor: (**a**) assembled view; and (**b**) exploded view containing (i) top clamp, (ii) bottom clamp, (iii) scaffold, (iv) linear actuator, (v) actuator support base (vi) fixture-1 actuator side, (vii) fixture-2 actuator side, (viii) movable plate, (ix) right-side guideway, (x) left-side guideway, (xi) force sensor, (xii) fixture-1 force sensor side, (xiii) fixture-2 force sensor side, (xiv) stationary plate, (xv) culture chamber, (xvi) base plate for culture chambers, (xvii) bioreactor mounting plate, (xviii) pedestal, (xix) bioreactor shield, and (xx) air filter.

The bioreactor components designed to achieve the key features are presented in Figure 5 and Table A1. As shown in Figure 5a, three culture chambers are parallelly assembled in the bioreactor for the simultaneous culture of three independent scaffolds; the exploded view (Figure 5b) shows only one bioreactor chamber for simplicity. Rigid clamping is achieved by designing a two-part clamp with pins and ridges on the gripping surface (Figure 5b(i,ii)). The scaffold (Figure 5b(iii)) is designed with appropriately located holes to assemble through the pins on the clamps. After locating the scaffold, the top and bottom of the clamps are to be firmly clamped using screws and nuts. For applying tensile stimulation, the clamps on one side of the scaffold are connected with the linear actuator (Figure 5b(iv)), fixed on the actuator support base (Figure 5b(v)), through a pair of fixtures (Figure 5b(vi,vii)) and a movable plate (Figure 5b(viii)) that can translate through a pair of linear guideways (Figure 5b(ix,x)). To evaluate the magnitude of force applied to the scaffold during mechanical stimulation, the clamps on the other side are connected to the force sensor (Figure 5b(xi)) through another pair of fixtures (Figure 5b(xii,xiii)) and fixed to the end of the guideways with a stationary plate (Figure 5b(xiv)) to prevent any movement. The culture chamber (Figure 5b(xv)) is designed with a media inlet and an outlet that can be connected to the media circulation system. The inlet and outlet are designed on the walls transverse to the linear motion to avoid back pressure on media due to the cyclic motion. The culture chambers are set on a base plate (Figure 5b(xvi)) to maintain their designated location on the bioreactor mounting plate (Figure 5b(xvii)). The linear actuator, actuator support base, guideways, and the base plate of the culture chambers are secured on the mounting plate with screws and nuts. The mounting plate is supported by pedestals (Figure 5b(xviii)) to reduce friction. All bioreactor components are covered inside a clear shield (Figure 5b(xix)) for protection against contamination and to maintain airflow only through the sterile air filter (Figure 5b(xx)).

**Figure 6:**
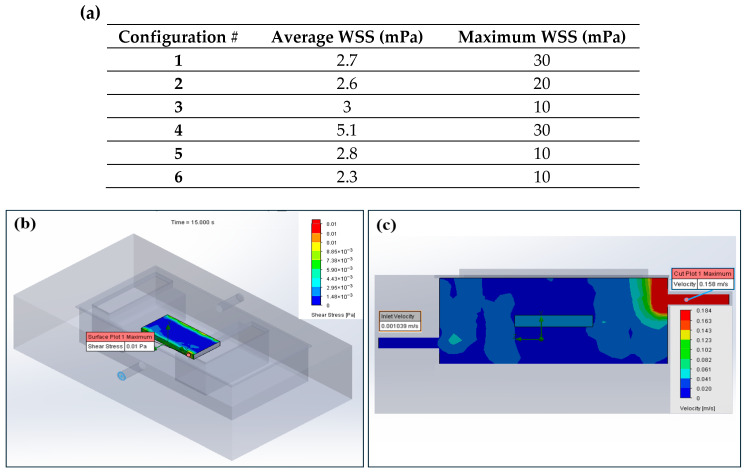
(**a**) Estimated WSS for different inlet/outlet configurations, (**b**) WSS profile on the scaffold surface, and (**c**) velocity profile for configuration 6.

**Figure A4:**
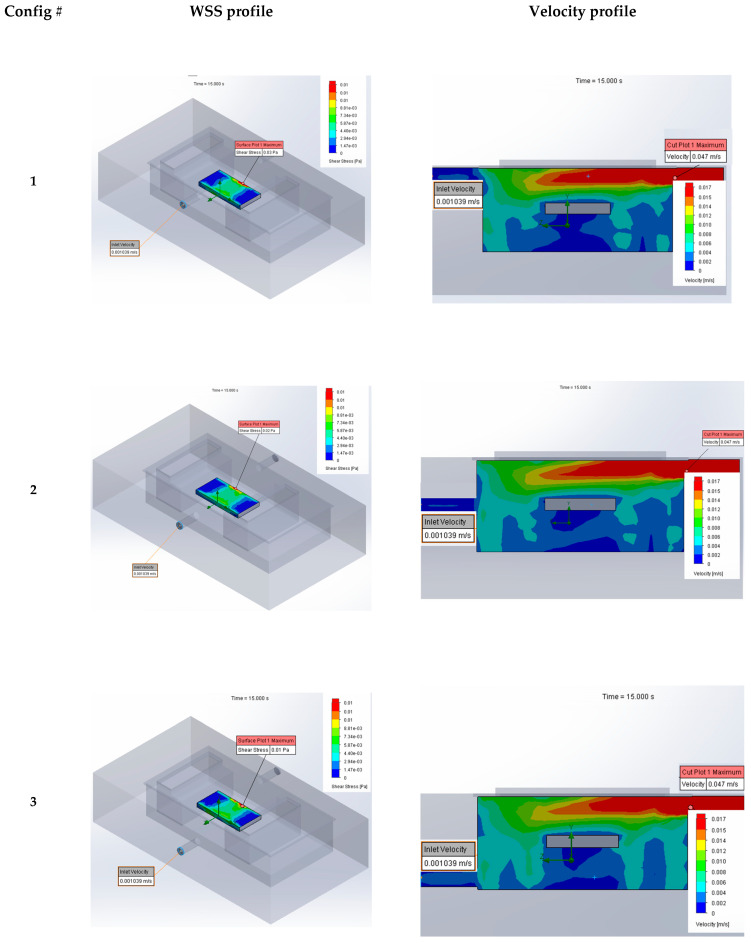

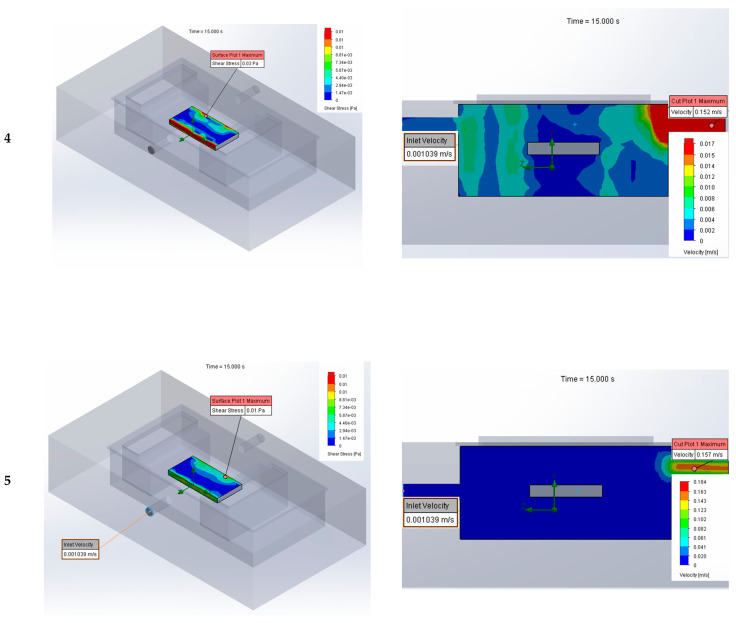
WSS profile on the scaffold surfaces and velocity profile along the cross-sectional plane through the inlet/outlet for configurations #1–5.

The estimated WSS values for the six different inlet/outlet configurations are shown in Figure 6a. The lowest average WSS was observed for configuration 6, where the inlet was positioned 2 mm below the scaffold and the outlet 2 mm above the scaffold. The WSS profile on the scaffold surface and the velocity profile on the cross-sectional plane through the inlet/outlet for configuration 6 are presented in Figure 6b,c, respectively. The WSS profiles and velocity profiles for the other configurations are shown in Figure A4.

The results are consistent with the findings from prior studies on WSS in perfusion bioreactors, which demonstrated that reducing the distance between the scaffold and the outlet contributes to a decrease in WSS [55]. Additionally, positioning the inlet below the scaffold has been shown to enhance the circulation of growth media through the culture chamber [56]. The maximum WSS for configuration 6 was also within the considerable range to avoid cell detachment from the scaffold surface [55,57]. Therefore, this configuration was selected for culture chamber fabrication.

**Figure 7:**
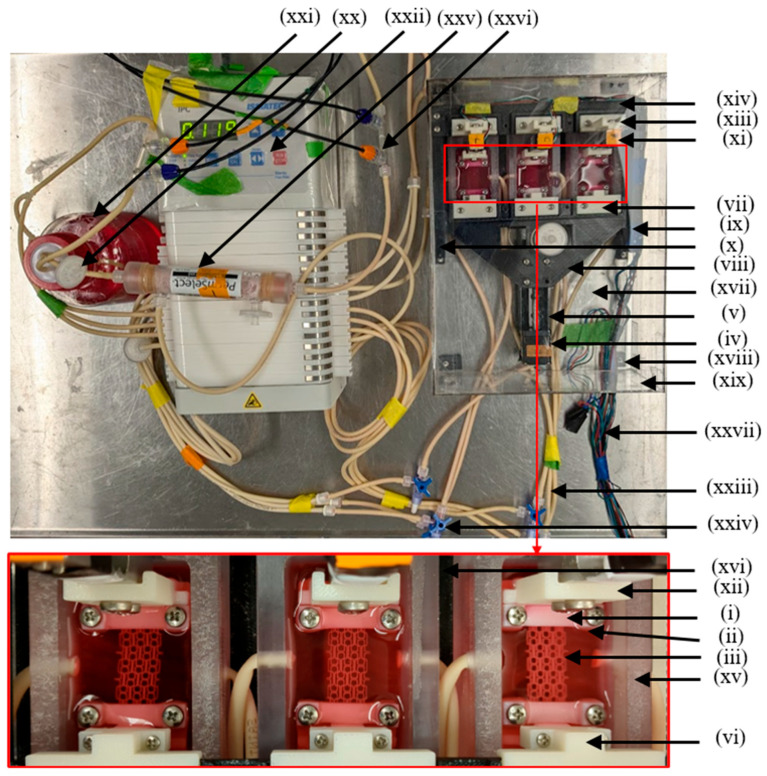
Bioreactor setup for perfusion and cyclic tensile stimulation on auxetic scaffolds: (i) top clamp, (ii) bottom clamp, (iii) scaffold, (iv) linear actuator, (v) actuator support base (vi) fixture-1 actuator side, (vii) fixture-2 actuator side, (viii) movable plate, (ix) right-side guideway, (x) left-side guideway, (xi) force sensor, (xii) fixture-1 force sensor side, (xiii) fixture-2 force sensor side, (xiv) stationary plate, (xv) culture chamber, (xvi) base plate for culture chambers, (xvii) bioreactor mounting plate, (xviii) pedestal, (xix) bioreactor shield, (xx) air filter, (xxi) media reservoir, (xxii) peristaltic pump, (xxiii) tube, (xxiv) valve, (xxv) hollow fiber silicone membrane gas exchanger, (xxvi) pH and DO sensors, and (xxvii) electric wires.

The assembled bioreactor system used in the rest of the study, including required additional standard components (Figure 2), is shown in Figure 7. To enable perfusion, the inlets and outlets of the culture chambers were connected to the media reservoir (Figure 7(xxi)) through a peristaltic pump (Figure 7(xxii)), using tubing (Figure 7(xxiii)) and valves (Figure 7(xxiv)). A hollow fiber silicone membrane gas exchanger (Figure 7(xxv)) was connected to the inlet to facilitate continuous oxygenation. Sensors (Figure 7(xxvi)) were also connected to the inlet for monitoring pH and DO in the growth medium. All components were sterilized using EtOH prior to assembly and aseptic techniques were employed to maintain sterility throughout the study.

### 3.2. Baseline Tensile Properties of PCL Auxetic Scaffold

**Figure 8:**
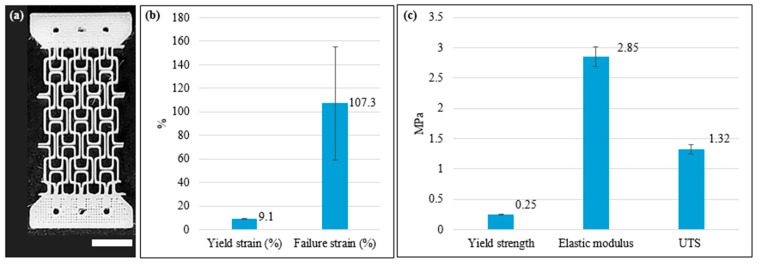
(**a**) A representative 3D-bioplotted PCL auxetic scaffold (scale bar = 6 mm) and (**b**,**c**) summary of baseline monotonic tensile properties (n = 3).

Figure 8 illustrates a representative 3D-bioplotted PCL auxetic scaffold and summarizes the monotonic tensile properties determined from the stress–strain data. The PR of the scaffold, negative as expected for an auxetic design, was −2.85 ± 0.01. These tensile properties fall within the physiological range of soft tissues such as heart, stomach, tendons, and skin [58]. As such, NPR is not inherent to typical biopolymers such as PCL, which makes these auxetic scaffolds particularly suitable for mimicking native tissues such as tendons and skin [32,33]. These scaffolds are also well-suited for engineering tissue patches for organs that naturally undergo expansion and contraction, including the heart, liver, and stomach [24,25]. These monotonic tensile properties are represented as the baseline for comparison with fatigue properties in Section 3.3.

### 3.3. Effects of Perfusion and Cyclic Tensile Stimulation on Scaffold Fatigue Properties

**Figure 9:**
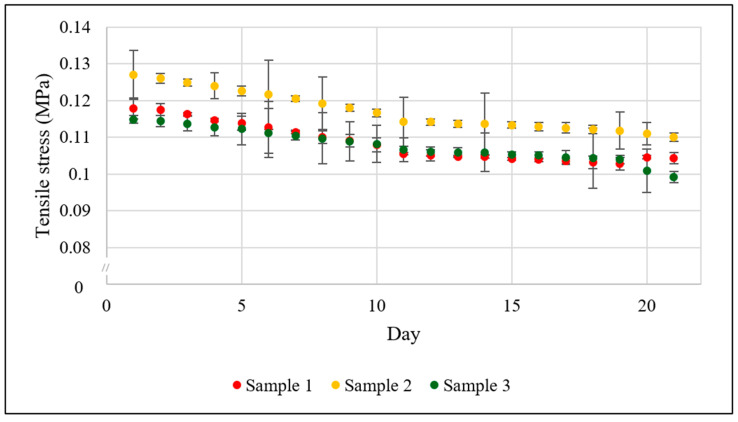
Trends in tensile stress (mean ± standard deviation over 2000 cycles/day) acting on the scaffolds (n = 3 independent samples, each tested individually) at 5% strain over 21 days of bioreactor culture due to the cyclic mechanical stimulation (cyclic strain = 0–5%) and perfusion (0.15 mL/min).

Figure 9 shows the trend in tensile stress (mean ± standard deviation over 2000 cycles/day) acting on each of the three scaffold samples (independently tested) due to the cyclic stimulation at the assigned strain rate (0–5%) over 21 days under perfusion. The applied tensile stress was reduced by 12.8 ± 1.2%, which can be attributed to material relaxation and structural adaptation under cyclic loading, where viscoelastic responses and microstructural realignment decreased the stress required to maintain the strain levels [20].

**Figure 10:**
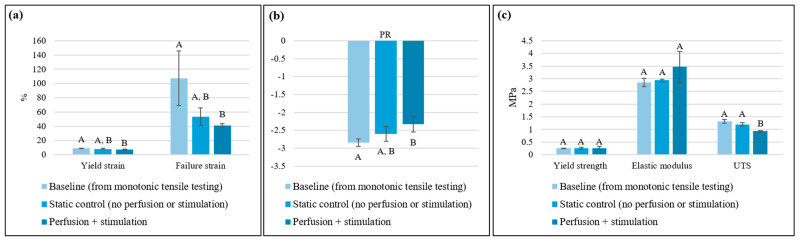
(**a**–**c**) Effects of perfusion and cyclic tensile stimulation on tensile properties of PCL auxetic scaffolds (n = 3/group). Data points which do not share a common letter are significantly different (*p* < 0.05). The baseline properties first reported in Figure 8. (**b**,**c**) are included for comparison.

The effects of perfusion and cyclic tensile stimulation on the tensile properties of the scaffolds after 21 days in PBS in the bioreactor compared to the baseline (i.e., monotonic properties of bioplotted scaffolds not subjected to static or dynamic conditions in PBS in the bioreactor; from Section 3.2) are summarized in Figure 10. The differences in the yield strain, failure strain, and PR between the perfusion + stimulation and static control groups were not statistically significant, indicating that the application of dynamic conditions over the 21 days in the bioreactor did not adversely affect these fatigue properties (Figure 10a,b). However, these properties for the perfusion + stimulation group were significantly lower than the baseline (*p* < 0.05). This could be attributed to the loss of ductility and resilience due to hydrolytic degradation and alterations in crystallinity during incubation of the scaffolds in PBS at 37 °C [59], which were exacerbated by the perfusion flow and cyclic loading. The dynamic conditions (perfusion + stimulation) in the bioreactor did not affect the scaffold yield strength or elastic modulus, but they resulted in a significantly lower UTS (*p* < 0.05) compared to static bioreactor conditions as well as the baseline (Figure 10c). Under cyclic strains, scaffold degradation can occur due to microstructural damage, stress concentration, and decreased efficiency during absorption and dissipation of strain energy [60,61], likely contributing to the observed reduction in UTS. As such, while UTS is one of the key parameters to consider during material selection and scaffold design, the scaffold yield strength and elastic modulus are functionally utmost critical for tissues subjected to regular cyclic loading, and it is important that these properties not degrade significantly in response to such loading.

### 3.4. Effects of Perfusion and Cyclic Tensile Stimulation on Cellular Responses

**Figure 11:**
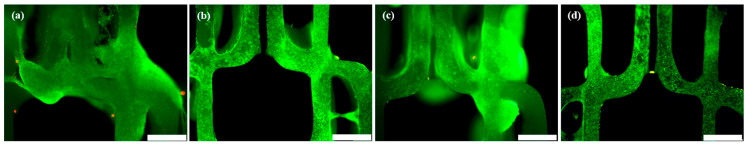
Live/dead images after 21 days of bioreactor culture from the groups under (**a**) perfusion + stimulation, (**b**) stimulation only (no perfusion), (**c**) perfusion only (no stimulation), and (**d**) static culture (no perfusion or stimulation) (magnification 2.5×; scale bar = 600 µm). Green indicates live cells, while red indicates dead cells.

**Figure 12:**
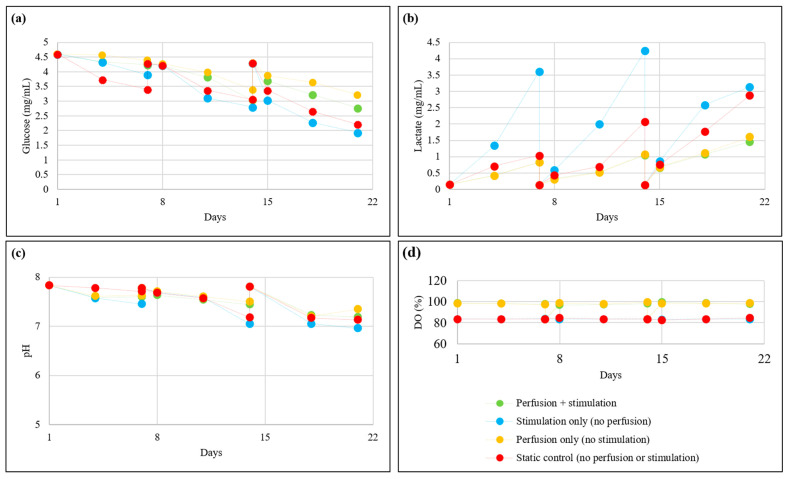
Effect of perfusion and tensile stimulation on (**a**) glucose concentration, (**b**) lactate concentration, (**c**) pH, and (**d**) DO during 21-day bioreactor culture.

**Figure 13:**
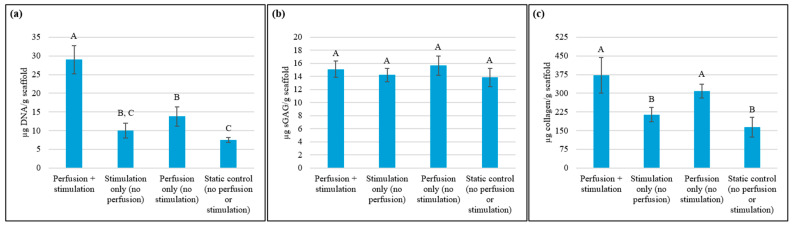
Effect of perfusion and tensile stimulation on (**a**) DNA, (**b**) sGAG, and (**c**) collagen content after 21-day bioreactor culture (data points which do not share a common letter are significantly different (*p* < 0.05); n = 3/group).

Four hours after the cell seeding procedure, the average seeded cell density was determined to be 2.8 × 10^5^ cells/scaffold. Given the large interstrand gaps in the geometric pattern of the missing-rib scaffolds, the low seeding density (reflecting the proportion of cells that actually adhered to the scaffold surfaces) was expected. Nonetheless, the results of the live/dead assay (Figure 11) highlighted substantial cell viability across all four groups—97.4 ± 0.6, 97.6 ± 1.6, 96.3 ± 1.5, and 94.9 ± 1.9% in the perfusion + stimulation, stimulation-only, perfusion-only, and static control groups, respectively—after 21 days of bioreactor culture, with cells displaying an elongated morphology indicative of healthy cellular function. The groups subjected to perfusion (perfusion + stimulation and perfusion-only) (Figure 11a,c) showed higher gap spanning, suggesting higher cell proliferation compared to the other two groups. In contrast, the static control group exhibited the least amount of gap spanning, indicating lower cell proliferation. The quantitative analyses on growth media conditions and cell proliferation are presented later in Figure 12 and Figure 13, respectively.

Figure 12a,b show the glucose and lactate contents and Figure 12c,d show the pH and dissolved oxygen (DO) in the growth media, respectively, during the 21-day bioreactor culture. Since perfusion enabled continuous medium circulation through the culture chamber, the groups cultured under perfusion maintained higher glucose levels and lower lactate levels closer to those in fresh media (refreshed at the end of days 7 and 14). The pH in these groups remained near that of the fresh growth media. Additionally, the DO levels in these groups were elevated due to continuous oxygenation. In contrast, the groups without perfusion exhibited a faster reduction in glucose and an accumulation of lactate in the medium, indicating reduced nutrient availability and increased waste buildup, which subsequently led to a reduction in pH. DO levels in these groups were lower than those with perfusion, reflecting lower oxygen diffusion in static media conditions.

Figure 13 summarizes the results from the biochemical assays. The perfusion + stimulation group exhibited significantly higher cellular DNA content than the other groups. The DNA content was—3.9 times higher than the static control (Figure 13a), reflecting the most substantial level of cell proliferation attributed to the synergistic effects of cyclic mechanical loading and perfusion. The perfusion-only scaffold group also significantly outperformed the stimulation-only and static control groups in terms of their DNA content. Mechanical stimulation promotes proliferation in NIH 3T3 fibroblasts by activating mechanotransduction pathways involved in the cell cycle, such as the upregulation of Egr1, Myc, and Fos, remodeling the cytoskeleton, and stimulating growth factor pathways [62]. Furthermore, perfusion culture ensures a stable nutrient supply and efficient waste removal, establishing an optimal environment for sustained cell proliferation [2,5,6,7,8]. Although tensile stimulation alone can stimulate proliferation, it cannot sustain prolonged proliferation in the absence of perfusion. Thus, the simultaneous application of perfusion and tensile stimulation is essential to achieve the highest level of proliferation.

The differences in sGAG content across the groups were not significant (Figure 13b), which suggests that sGAG synthesis in NIH 3T3 cells was minimally responsive to mechanical and perfusion stimuli within the PCL auxetic scaffold. This minimal response may be attributed to fibroblast phenotype, as these cells primarily favor fibrillar ECM depositions, such as collagen and fibronectin [63], over the proteoglycan-rich matrix typical of chondrocytes [64]. In addition, although sGAG production in fibroblasts can be increased by mechanical stimulation [65], sGAG synthesis is also influenced by factors such as scaffold matrix stiffness [66] and the presence of biochemical cues [67]. Herein, the relatively high stiffness and hydrophobic nature of PCL may be limiting the availability of biochemical motifs and compliance required to support pericellular matrix formation and proteoglycan retention. Whether softer biomaterials with natural biochemical motifs, such as hydrogels, instead of synthetic thermoplastics like PCL, and alternative culture conditions can enhance sGAG production in auxetic scaffolds by providing a more compliant matrix environment is an important topic for future research.

The groups under perfusion culture showed significantly higher collagen content compared to the stimulation-only and static control groups (Figure 13c). The perfusion + stimulation and the perfusion-only groups secreted 2.3 and 1.9 times more collagen, respectively, on average, compared to the static control. Perfusion plays a key role in supporting collagen synthesis by enhancing nutrient delivery and waste removal, both critical for extracellular matrix production. Tensile stimulation by itself did not lead to improvement in collagen content compared to the static control. Future research should determine whether higher levels of cyclic strain can lead to increased collagen secretion [68,69].

Taken together, the results of this study highlight the function of the custom bioreactor and complementary roles of perfusion and mechanical cues in enhancing scaffold biofunctionality. Notably, the scaffolds maintained mechanical integrity under perfusion and 0–5% cyclic strain, and these dynamic conditions also resulted in superior cell proliferation and collagen content by enhancing nutrient transport, waste removal, oxygenation, and mechanical signaling. The current sample size (n = 3) and 21-day terminal endpoint were selected to establish a foundational baseline for the comparative validation of the bioreactor’s multi-modal capabilities using a representative auxetic scaffold design. While standardized material testing protocols (e.g., ASTM D638) typically suggest larger sample sizes for absolute characterization, the trends identified here provide a necessary framework for in-depth future investigations. Such work could utilize broader bioprocessing parameter ranges, increased sample sizes to enhance statistical power, and longitudinal sampling to characterize temporal growth kinetics. Furthermore, these studies may employ advanced morphological assessments to explore how multi-modal stimulation influences cell adhesion and directional alignment within diverse auxetic geometries. Expanding these investigations across a wider array of biomaterials under both tensile and compressive loading will facilitate the optimization of scaffold designs and culture conditions for tissue-specific applications such as tendon, skin, and cardiac muscle. Ultimately, this versatility, combined with the ability to tune dynamic culture parameters, positions the bioreactor system as a robust tissue engineering platform.

## 4. Conclusions

A new multi-modal perfusion–tensile stimulation bioreactor was developed and used to study effects of the dynamic culture conditions on the mechanical properties and cellular responses of 3D-bioplotted PCL missing-rib auxetic scaffolds over 21 days. The bioreactor design ensured minimal WSS to support cell attachment and proliferation on the scaffold surfaces, while the mechanical transmission system enabled controlled cyclic motion and in situ stress measurement.

This is the first study in the literature to demonstrate the synergy between perfusion and mechanical stimulation in promoting biofunctional responses in auxetic scaffolds characterized by NPR. Scaffolds subjected to perfusion alone exhibited suboptimal cell proliferation due to the absence of mechanical cues required for effective mechanotransduction, while tensile stimulation by itself failed to sustain cell proliferation and ECM secretion due to inadequate nutrient and oxygen supply. The missing-rib auxetic scaffold examined herein supported cell proliferation and collagen secretion under 0–5% cyclic strain and demonstrated tensile fatigue properties and NPR suitable for musculoskeletal applications.

While this study established a foundational baseline using a representative missing-rib design, the bioreactor system’s versatility enables future exploration of broader design spaces. Future work can expand the range of bioprocessing parameters, incorporate longitudinal time points, investigate diverse auxetic geometries, and utilize larger sample sizes for more rigorous biofunctional and statistical characterization. Such investigations will allow for in-depth morphological analyses to optimize the mechanobiological environment for specific tissue engineering applications, including tendon, skin, and cardiac muscle.

## Figures and Tables

**Table 1:** Bioreactor culture parameters for characterizing the effects on fatigue properties of the scaffold.

Parameter	Level
Frequency	0.1 Hz
Applied strain	0–5%
No. of cycles/day	2000
Total number of cycles in 21 days	42,000
Media circulation rate	0.15 mL/min

## Data Availability

The raw data supporting the conclusions of this article will be made available by the authors on request.

## Appendix A

**Table A1:** Bill of materials for fabricated components used in the bioreactor system.

Component Name	# of Units Used in the Bioreactor	Material	Fabrication Process
Top clamp	6	Acrylonitrile butadiene styrene (ABS) (Stratasys Ltd., Minneapolis, MN, USA)	Extrusion 3D printing (Stratasys F120, F370, Stratasys Ltd.)
Bottom clamp	6		
Actuator support base	1		
Fixture-1 actuator side	3		
Fixture-2 actuator side	3		
Movable plate	1		
Right-side guideway	1		
Left-side guideway	1		
Fixture-1 force sensor side	3		
Fixture-2 force sensor side	3		
Stationary plate	1		
Base plate for culture chambers	1		
Pedestal	4		
Culture chamber	3	FDA-grade polycarbonate block (Plasti-Block^®^, Omachron Plastics Inc., Pontypool, ON, Canada)	CNC machining (VF-2 Machining Center, Haas Automation, Inc., Oxnard, CA, USA)
Bioreactor mounting plate	1	Acrylic sheet (thickness: 1/8 in)	Laser cutting (Glowforge Inc., Seattle, WA, USA)
Bioreactor shield	1		

**Table A2:** Details of PicoGreen, OHP, and DMMB assays.

Assay	Method
PicoGreen	The Quant-iT™ PicoGreen dsDNA Assay (Thermo Fisher Scientific, Waltham, MA, USA) was used for DNA quantification. Standards of known DNA content (0 to 1 ng/µL) were prepared using Lambda DNA standard (Thermo Fisher Scientific) to obtain a standard curve, from which DNA contents of tested samples were interpolated. A total of 2 µL of each digested sample was diluted in 98 µL of nucleus-free H_2_O to produce a 1:50 dilution. A total of 10 µL of each standard or diluted sample was added in triplicates in a black 96-well plate. In a dark room, 100 µL of PicoGreen working reagent solution (0.5% PicoGreen reagent, 5% TE buffer, and 94.5% ddH_2_O) (Thermo Fisher Scientific) was added to each well and incubated for 5 min. After incubation, fluorescence intensity was measured using a microplate reader (Infinite 200, Tecan, Männedorf, Switzerland) with excitation and emission wavelengths of 480 and 520 nm, respectively, to determine the total DNA content.
OHP	The standards containing known concentrations of OHP (0 to 800 μg/mL) were prepared using Trans-4-OHP (Sigma-Aldrich, St. Louis, MO, USA) to obtain a standard curve for subsequent interpolation of OHP content in the test samples. A total of 50 μL of each standard or digested sample was subjected to hydrolysis in 100 μL of 4 N NaOH at 120 °C for 20 min. The samples were neutralized with 100 μL of 4 N HCl. The standards and samples were then incubated in 600 µL of Chloramine-T solution (Sigma-Aldrich) at room temperature for 20 min, followed by incubation in 600 µL of Ehrlich’s solution (Sigma-Aldrich) at 65 °C for 20 min. Triplicates of 200 µL from each standard or sample were dispensed in a transparent 96-well plate, and the absorbance was measured at 540 nm using the microplate reader to quantify the total OHP content. The total collagen content was calculated as 7.5 times the OHP content.
DMMB	The standards of known sGAG content (0 to 50 μg/mL) were prepared using shark chondroitin-6-sulfate (Sigma-Aldrich) to obtain a standard curve, from which sGAG contents of the test samples were interpolated. A total of 40 μL of each standard or digested sample was added in triplicates in a transparent 96-well plate. The DMMB dye solution was prepared by combining 1.25 mL of a stock solution (16 mg DMMB (Sigma-Aldrich) dissolved in 5 mL of ethanol) with 250 mL of an aqueous solution adjusted to pH 1.5. The aqueous solution contained 0.5 mg of sodium formate, 0.5 mL of formic acid, and 2–3 mL of HCl to achieve the desired pH. In a dark room, 250 μL of the DMMB dye solution was added to each well, and absorbance was measured at 540 and 600 nm using the microplate reader within 5 min of adding the dye to determine the total sGAG content.
